# Long-term SARS Coronavirus Excretion from Patient Cohort, China

**DOI:** 10.3201/eid1010.040297

**Published:** 2004-10

**Authors:** Wei Liu, Fang Tang, Arnaud Fontanet, Lin Zhan, Qiu-Min Zhao, Pan-He Zhang, Xiao-Ming Wu, Shu-Qing Zuo, Laurence Baril, Astrid Vabret, Zhong-Tao Xin, Yi-Ming Shao, Hong Yang, Wu-Chun Cao

**Affiliations:** * Beijing Institute of Microbiology and Epidemiology, Beijing, People's Republic of China;; † Institut Pasteur, Paris, France;; ‡ Caen University, Paris, France;; § Beijing Institute of Basic Medical Sciences, Beijing, People's Republic of China; and; ¶ Chinese Center for Disease Control and Prevention, Beijing, People's Republic of China

**Keywords:** Dispatch, Severe acute respiratory syndrome, epidemiology, cohort, transmission, diagnosis, RT-PCR

## Abstract

This study investigated the long-term excretion of severe acute respiratory syndrome–associated coronavirus in sputum and stool specimens from 56 infected patients. The median (range) duration of virus excretion in sputa and stools was 21 (14–52) and 27 (16–126) days, respectively. Coexisting illness or conditions were associated with longer viral excretion in stools.

Severe acute respiratory syndrome (SARS) is a newly emerged disease that spread globally in early 2003; more than 2,523 cases and 181 deaths occurred in Beijing, China (*1**–**3*). In the initial outbreak period, the diagnosis of infection was mostly dependent on clinical manifestations and epidemiologic findings, until the associated coronavirus (SARS-CoV) was identified as the causal agent (*4**–**6*), and its immediate full genome was sequenced (*7**,**8*).

Molecular assays using reverse transcriptase–polymerase chain reaction (RT-PCR) were developed as an important diagnostic tool on a variety of clinical samples (*4**–**6**,**9*). Several studies have explored the optimal source and timing of sample collection for SARS diagnosis on the basis of RT-PCR results (*5**,**10**–**12*). While viral genetic material could be detected by RT-PCR in stools for up to 50 days, no virus could be cultured after 3 weeks of illness from corresponding specimens (*12*). Although long-term viral excretion has important public health implications through its potential spread of the virus to the environment, no study has yet explored factors associated with prolonged viral excretion and risk for transmission to household contacts. Our study was designed to investigate prospectively the viral shedding from a cohort of Beijing SARS patients for up to 6 months. Clinical and epidemiologic data were used to identify the potential risk factors for prolonged virus shedding. Household contacts of SARS patients were clinically monitored for secondary transmission.

## The Study

Patients were recruited for the study at one of the designated hospitals for SARS patients in Beijing. The diagnosis of probable SARS was made in accordance with the definition promulgated by the World Health Organization. Briefly, the case definition included the following: fever of >38°C, cough or shortness of breath, new pulmonary infiltrates on chest radiography, and a history of exposure to a patient with SARS or absence of response to empirical antimicrobial therapy for typical and atypical pneumonia. Stool and sputum specimens from probable SARS patients were collected weekly while they were in the hospital and monthly after they were discharged, up to 6 months after onset of disease. Specimens were transported in viral transport medium and stored at –70°C until testing.

After informed consent was obtained, epidemiologic and clinical data were collected from all study participants by using a standard data collection form, through interview and medical record review. The obtained information included the following items: age, sex, occupation, medical history, time and nature of exposure, symptoms and physical findings, laboratory tests at admission to the hospital, and outcomes on discharge. A questionnaire was also used to identify any suspected or probable SARS case among household contacts of SARS patients after they were discharged. The questionnaire was administered 1 month after discharge from the hospital, and at the end of the followup period.

Specimens were analyzed after the RT-PCR protocol was optimized in the laboratory. Early samples from all patients were tested, and only patients with at least one sample that tested positive for SARS-CoV RNA during the first 16 days were kept in the study. All subsequent samples from the same patients were then tested until three consecutive samples were negative; the first negative sample defined the time of negativation. RNA from clinical samples was extracted by using the QIAamp virus RNA mini kit (Qiagen, Hilden, Germany), as instructed by the manufacturer. Total RNA was then reverse transcribed with random hexamers, and cDNA was amplified with two primer pairs. The first pair, COR-1/COR-2, is one of the primer pairs recommended by the World Health Organization (http://www.who.int/csr/SARS/primers/en/). The second pair, R1/R2, was designed to amplify a 220-bp fragment of the replicase gene (R1: 5´ AGG TTA GCT AAC GAG TGT GCG 3´, R2: 5´AGC CTG TGT TGT AGA TTG CGG 3´). These two primer pairs, COR-1/COR-2 and R1/R2, had been selected among others (including Cor-p-F2/Cor-p-R1, Cor-p-F3/Cor-p-R1, SAR1s/SAR1as) for their higher sensitivity (87% and 95%, respectively), using serologic testing as the standard. Congruence between the results of the two tests was 86% (F. Tang, pers. comm.). Only specimens with positive results for both primer pairs were considered positive, so that false-positive results were eliminated. During this procedure, positive and negative controls were systematically included and treated in the same way as the virus sample.

Viral isolation was performed on RT-PCR–positive stool samples from convalescent patients only. The sample was pretreated by centrifugation, and the supernatant was injected after filtration into the VeroE6 cell culture at 37°C. All procedures were handled under biosafety level 3 conditions. We looked for the characteristic cytopathic effect for 5 to 11 days after injection. Examination with electron microscope and RT-PCR assays were used for identification of SARS-CoV.

All data were processed by SPSS (SPSS Inc. Chicago, IL) and Stata (Stata Corporation, College Station, TX) software. Categorical and continuous variables were compared across study groups by means of χ^2^, Fisher exact test, and Mann-Whitney U tests, where appropriate. Survival analysis was performed by using time to RT-PCR negativation as the outcome variable (for stool and sputum specimens separately). Survival curves were built by using the Kaplan-Meier method. Correlation coefficient between time to RT-PCR negativation in stools and sputum was calculated by using the Spearman rank correlation coefficient.

From mid-March to early May 2003, a total of 83 probable SARS patients were admitted to a Beijing hospital dedicated to SARS patients. Of these, 56 (67.4%) had at least one RT-PCR–positive sample in the first 16 days of hospitalization and were therefore enrolled in the study. The study group consisted of 31 male and 25 female participants, with a median age of 31 and 34 years, respectively. Of the 56 patients, 21 (37.5%) were healthcare workers. The median (range) duration between onset of symptoms and hospitalization was 2 (0–6) days, and the median (range) duration of hospital stay was 32 (21–58) days. Six patients had coexisting medical conditions or illnesses at enrollment or during followup: diabetes (2 patients), heart disease (2 patients), pulmonary tuberculosis (2 patients, including 1 with diabetes), and high blood pressure (1 patient). No patient died.

A total of 514 stool and 493 sputum specimens were collected from the 56 patients during the study period. The median (range) duration between onset of symptoms and first positive RT-PCR test result was 6 (3–10) days for stool and 6 (3–16) days for sputum. RT-PCR sputum and stool specimens did not show negative results until day 14 and day 16 after disease onset, respectively (Figure). The median (range) duration of viral excretion was 21 (14–52) days for sputum samples, and 27 (16–126) days for stool specimens. Negative RT-PCR results occurred at the same time in sputum and stool samples for 45 patients, and at a later time in stool samples than in sputum in 11 patients (the Spearman rank correlation coefficient between time to RT-PCR negativation in sputum and stool was 0.65, p < 0.0001). Four patients had viral excretion in stools after 100 days; three of these patients had coexisting conditions, such as pulmonary tuberculosis (2 patients), diabetes (1 patient, who also had tuberculosis), and high blood pressure (1 patient). The proportion of patients with coexisting conditions was significantly higher for patients with viral excretion >100 days compared to others (3/4 vs. 3/52, p = 0.003, Fisher exact test). All attempts (n = 12) to isolate virus from RT-PCR–positive stool specimens collected >6 weeks after disease onset failed. All 56 patients had at least one close contact. Clinical symptoms suggestive of SARS did not develop in any of the 70 close contacts of convalescent SARS patients we surveyed.

**Figure:**
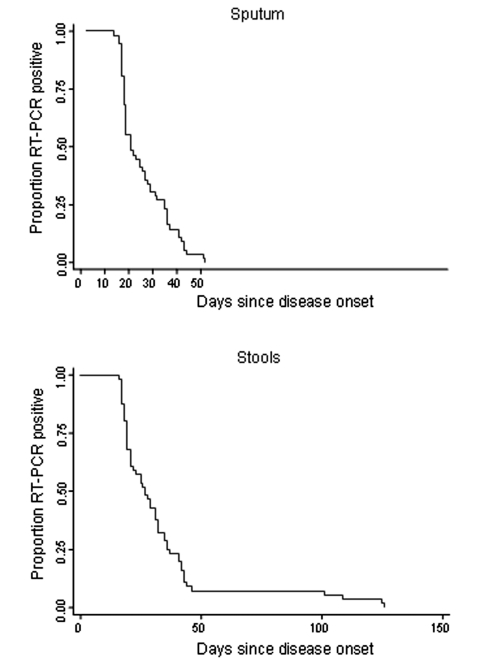
Time lapse before sputum and stool specimens of patients with severe acute respiratory syndrome (SARS) (n = 56) were negative by reverse transcription-polymerase chain reaction, at one designated SARS hospital, Beijing, 2003.

## Conclusions

This study is the first to document duration of SARS-CoV excretion in sputum and stool in a cohort of SARS patients. The median duration of viral excretion was 3 weeks in sputa and 4 weeks in stool samples. This estimate might be high, since some patients with no or short viral excretion might have been missed because of the study design. In most patients (45 of 56), the duration of viral excretion in both specimens was identical, while it was longer in stool samples compared to sputa for 11 patients. The long duration of excretion (>100 days after onset of symptoms) observed in stools of four patients suggests that independent replication of virus may take place in the intestinal tract. Three of these four patients had coexisting conditions, a known risk factor for prolonged SARS illness (*13**,**14*). Prolonged viral excretion may have important public health implications if responsible for spread of the virus to other persons or to the environment. Two findings of this study are reassuring in this regard: one is the failure of all attempts to isolate the virus from positive RT-PCR specimens collected in convalescent patients, which suggests that excreted virus was no longer infectious; the other is the absence of SARS in close contacts of convalescent patients during the study period.

Our study has demonstrated long-term duration of SARS-CoV excretion in stools. Careful hand and toilet disinfection is required to avoid transmission of the virus to close contacts of patients.
